# Appetitive and Dietary Effects of Consuming an Energy-Dense Food (Peanuts) with or between Meals by Snackers and Nonsnackers

**DOI:** 10.1155/2011/928352

**Published:** 2011-01-22

**Authors:** A. A. Devitt, A. Kuevi, S. B. Coelho, A. Lartey, P. Lokko, N. Costa, J. Bressan, R. D. Mattes

**Affiliations:** ^1^Department of Nutrition Science, Purdue University, 212 Stone Hall, West Lafayette, IN 47907-2059, USA; ^2^CSIR-Food Research Institute, P.O. Box M20, Accra, Ghana; ^3^Departamento de Nutrição e Saúde, Universidade Federal de Viçosa, Avenida PH Rolfs, s/n, 36570-000 Viçosa, MG, Brazil; ^4^Department of Nutrition and Food Science, University of Ghana, P.O. Box LG25, Legon, Ghana

## Abstract

*Background*. Energy-dense foods are inconsistently implicated in elevated energy intake (EI). This may stem from other food properties and/or differences in dietary incorporation, that is, as snacks or with meals. *Objective*. Assess intake pattern and food properties on acute appetitive ratings (AR) and EI. *Design*. 201 normal and overweight adults consuming a standard lunch. Test loads of 1255.2 kJ (300 kcal) were added to the lunch or provided as snack. Loads (peanuts, snack mix, and snack mix with peanuts) were energy, macronutrient, and volumetrically matched with a lunch portion as control. Participants completed meal and snack sessions of their randomly assigned load. *Results*. No differences were observed in daily EI or AR for meal versus snack or treatment versus control. Consumption of peanuts as a snack tended to strengthen dietary compensation compared to peanuts or other loads with a meal. *Conclusions*. Inclusion of an energy-dense food as a snack or meal component had comparable influence on AR and EI. Peanuts tended to elicit stronger dietary compensation when consumed as a snack versus with a meal. If substantiated, this latter observation suggests that properties other than those controlled here (energy, macronutrient content, and volume) modify AR and EI.

## 1. Introduction

Over the past 30 years, per capita daily energy intake has significantly increased in all segments of the US population [1].This has been attributed to increased energy intake within eating occasions and an increase in the number of daily eating occasions [2]. These dietary changes are reported contributors to the rising prevalence of obesity [3], but their etiology and consequences have not been fully characterized.

Increased energy intake at a single eating occasion may occur through the consumption of larger portion sizes [4–6] and/or the consumption of more energy-dense foods [7–12]. Study of the latter has yielded evidence that ingestion of high-fat and/or energy-dense foods promotes elevated chronic energy intake and body weight [7, 8, 10–20].However, a striking example of an inconsistent relationship involves nuts, one of the most energy-dense food categories. Epidemiological studies indicate that the inclusion of nuts in the diet, even at high levels, does not promote weight gain while controlled studies indicate that nuts can be effectively substituted for other sources of dietary energy [21–28]. This has been explained by their high satiety value, potential to increase energy expenditure, and the limited bioaccessibility of the fat they contain [29]. The former is the predominant contributor, raising mechanistic questions about the satiety effects of nuts. Factors that may be responsible include intrinsic properties of nuts such as their macronutrient composition, fiber content, or texture. Each of which was examined in the current protocol. 

The second ingestive behavior that could be responsible for the noted elevation of total daily energy intake is an increase in the number of daily eating occasions (“snacking”). Snacking stems, partly, from the increasing availability of single-serving products [5, 30] and has been documented to occur among all age groups [2]. Snacking has become so ubiquitous that the term “fourth meal” is gaining popularity in “pop culture” within the US [31]. However, the relationship between the number of eating occasions and BMI remains unclear with studies reporting a direct, inverse, or no association [32–41]. These inconsistent reports may be due to methodological issues, among them, differences in the macronutrient content of test foods [17, 42–46], deviations from habitual eating patterns [47, 48], and inconsistent definition of snack and meal within the literature [49, review of associated putative mechanisms]. To further understand the influence of intake pattern on overall energy intake, the current protocol assessed nut intake at both meal and snack eating occasions. 

The current study focused on peanuts because, in the US and abroad, peanuts are the most widely consumed nut (conventionally defined since they are technically a legume) [50]. Further, peanuts are an important dietary component for developing countries such as Ghana and Brazil, providing an inexpensive, energy-dense source of nutrients. As this is the case, a clear understanding of peanut influences on appetite and energy intake and potential cross-cultural differences is warranted and was performed here. Furthermore, peanuts are a particularly novel food in that they are consumed in all 3 countries both with meals and as snacks and provide high-energy density along with high nutrient density (e.g., protein, unsaturated fat, fiber, folate, magnesium, and phytonutrients such as resveratrol [51, 52]) and are crunchy and salty. Indeed, their beneficial effect on serum lipids was the basis for their inclusion in a US qualified health claim relating to reduced risk of cardiovascular disease [53]. When identifying common snack foods, those that are crunchy and salty account for approximately 40% of snack food ingestion in US adults [54].Currently, snack food intake preferences have not been characterized in Brazil or Ghana. Salty snacks, in particular, contribute significantly to increased energy intake [55]. Of these, the most common are fried chips and pretzels, not nuts [55]. 

An improved understanding of how peanut ingestion patterns may influence acute daily energy intake and appetitive responses can provide insights on the lack of effect of peanut intake on energy balance by providing detail related to the energy compensation mechanism that likely occurs with peanut intake. This would allow for the development of appropriate public health recommendations regarding their inclusion in the diet as well as other items with similar properties (e.g., other nuts or selected snack mixes). 

Due to the documented energy compensation that occurs with all nut, including peanut ingestion (approximately 65–75% of nut energy; [29]), it is expected that this work will provide evidence on which of the intrinsic properties of peanuts elicit this effect. Regarding intake at specific eating occasions, the peanuts would be consumed solely during the snack eating occasion, and thereby any intrinsic properties would presumably have full potency as compared to being attenuated during consumption with a mixed meal.As such, it is hypothesized that consumption during the snack eating occasion would reduce appetite and energy intake thereby demonstrating enhanced energy compensation compared to ingestion with a meal. 

## 2. Methods

### 2.1. General Protocol

Participants were recruited through three institutions: Purdue University in West Lafayette, IN, USA, the Food Research Institute, in Accra, Ghana, and the Federal University of Viçosa, in Brazil. Participants were recruited by public advertisement and were enrolled if they met eligibility criteria including good health, 18–50 y/o, not taking medication (except for birth control), consistent activity level and not an extreme athlete, no significant weight fluctuations (±5  pounds) within the last six months, nonsmoker, control over the purchase and preparation of at least 50% of their food, no allergy to peanuts, low dietary restraint [56], and consuming at least 3 meals per day with breakfast occurring between 6 AM–9 AM, lunch from 11 AM–1 PM, and dinner between 5 PM and 8 PM. In addition, subjective palatability ratings of the study foods, determined during screening, were >5 on a 9-point scale and ratings of the test loads (peanut, snack mix and snack mix with peanuts) did not differ by more than 2 units on the same scale. The participant's habitual snacking patterns were also assessed by subject self-report on frequency and timing of snack intake. This was used as eligibility criteria such that half were consumers of a mid-afternoon snack (i.e., self-report of an eating event between the hours of 2–4 PM on a typical day), and half were not habitual consumers of a mid-afternoon snack. 

Eligible participants were randomly assigned to one of three treatment arms defined by the provided test foods (loads): peanuts, snack mix, or snack mix with peanuts. Randomization to treatment occurred at each study location. In addition to their assigned treatment session, all participants completed a control session where an iso-energetic portion of the experimental lunch was also used as a test load. This control was chosen to allow for comparison of the treatments to a snack of differing macronutrient composition while maintaining equal compulsory energy intake throughout the study. The control or intervention foods were each presented at two eating occasions (with a lunch meal, “meal” or alone 120 minutes after the provided lunch, “snack”) on separate days. Thus, there were a total of four test days (Table 1). The lunch meal was comprised of a ham and cheese sandwich, carrot sticks, chocolate, and 400 g of water to drink (Table 2). This lunch meal provided a macronutrient composition of 34% fat, 16% protein, and 50% carbohydrate. Energy provided via the standard lunch was individualized to provide 30% of each individual's estimated total energy requirement assuming an activity factor of 1.5 [57]. Meals were provided near (±1  hour) the participants' customary lunchtime. The total energy provided from the control and treatment loads was held constant at 1255.2 kJ (300 kcal; Table 3). All test loads were provided with a 400 mL portion of water to drink, which was consumed in its entirety. The participants also completed appetitive questionnaires throughout each study session. 

### 2.2. Study Session Protocol

Participants reported to the laboratory on four separate days, approximately one hour prior to their habitual lunch meal time having consumed the same customary (for them) breakfast followed by a fast of ≥3.5 hours. Following baseline data collection, participants ingested the entire lunch meal within 15–30 minutes, half of the time with a test load per randomization schedule. For the other half of the trials, a test load was provided as a snack 120 minutes after meal initiation and was consumed in its entirety within 15 minutes. Questionnaires were completed for subjective ratings of hunger and fullness at times 0, 15, 30, 60, 120, 135, 150, 180, 240, and 300 min. 

### 2.3. Dietary Analysis

To obtain free-living dietary intake data, participants were verbally instructed on the completion of accurate diet logs. These logs were collected and analyzed using nutrient database software specific to each country. Total daily energy intake was estimated by including all food items ingested for the 24-hour period from 12 AM on the morning of a testing day through 11:59 PM. 

### 2.4. Appetitive Ratings

Subjective appetitive ratings were obtained in response to the following questions. (1) How strong is your feeling of hunger? (2) How strong is your feeling of fullness? The visual analog response scales were anchored with “Not at all” and “Extremely”. Ratings were quantified by the distance, in millimeters, from the low anchor point to the participant's mark.

### 2.5. Statistics

A mixed model ANOVA was used to assess differences in mean daily energy intake and appetitive ratings by load and meal. Additionally, mean appetitive ratings for each assessment time were calculated by load and meal then were analyzed with a mixed model ANOVA. Tukey's least significant difference test was computed, when appropriate, for post hoc analyses. Energy compensation scores were calculated as(({predicted  energy  requirement + 1255.2 kJ  load} − test  session  energy  intake)/1255.2 kJ  load)∗100. 

### 2.6. Statement of Ethics

I/we certify that all applicable institutional and governmental regulations concerning the ethical use of human volunteers were followed during this research. The research protocol was approved by the Institutional Review Boards at Purdue University, Food Research Institute, and Federal University of Viçosa.

## 3. Results

### 3.1. Participant Characteristics

A total of 201 participants from three countries (Brazil = 60, Ghana = 78, and United States = 63) were enrolled. Participants from each country were randomized to each of the three treatment arms with 22 Americans, 24 Ghanaians, and 20 Brazilians completing the peanut arm; 21 Americans, 27 Ghanaians, and 20 Brazilians completing the snack mix arm; and 20 Americans, 27 Ghanaians, and 20 Brazilians completing the snack mix with peanuts arm. The mean participant BMI was 22.9 ± 3.2 kg/m^2^ for Brazilians, 21.9 ± 2.0 kg/m^2^ for Ghanaians, and 23.1 ± 2.5 kg/m^2^ for Americans. A comparison of BMIs between counties revealed a significant difference (*F*(2,201) = 3.626, *P* = 0.028). However, this difference was not confirmed in post hoc analyses. Due to the small absolute difference in BMI and the lack of differences noted in energy intake between countries (Brazil = 2481.4 ± 78.9; Ghana = 2359.5 ± 63.6 and United  States = 2534.36 ± 71.3; *F*(2,587) = 1.79, *P* = 0.1683), energy intake from each country was pooled for analyses. There were 100 females and 101 males and the mean age of study participants was 24 ± 4 y for Brazilians, 25 ± 5 y for Ghanaians, and 22 ± 3 y for Americans. Ninety-six participants did not habitually eat mid-afternoon snacks and 105 did. 

### 3.2. Energy Intakes

**Figure 1:**
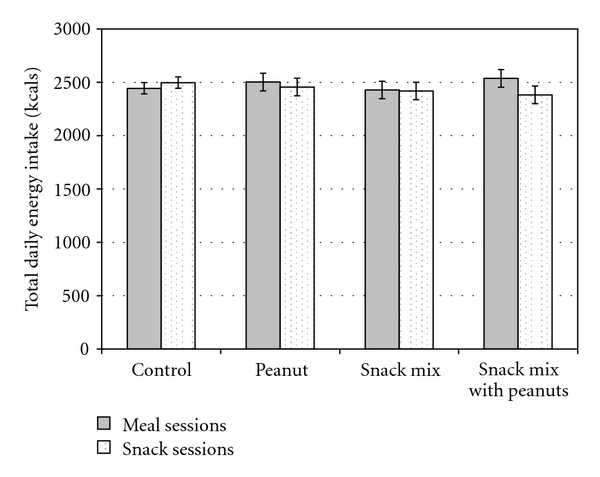
Mean total daily energy intake (SE) for each treatment and time combination. Peanut *n* = 66; Snack Mix *n* = 68; Snack Mix with Peanut *n* = 67; Control *n* = 201.

Total daily energy intake was not influenced by treatment with 10339 ± 188 kJ (2471 ± 45 kcal) ingested during control sessions, 10376 ± 276  (2480 ± 66 kcal) during peanut sessions, 10142 ± 276 kJ (2424 ± 66 kcal) during snack mix sessions, and 10293 ± 276 kJ (2460 ± 66 kcal) during snack mix with peanut sessions (*F*(3,587) = 0.21; *P* = 0.89). The time at which the provision was consumed did not have a significant influence on total daily energy intake with 10368 ± 197 kJ (2478 ± 47 kcal) being consumed during meal sessions and 10205 ± 197 kJ (2439 ± 47 kcal) consumed during snack sessions (*F*(1,587) = 0.75; *P* = 0.39). The ingestion of peanuts, snack mix, snack mix with peanuts and control resulted in similar total daily energy intakes across treatments and between meal and snack sessions (Figure 1; *F *(3,587) = 1.17; *P* = 0.32).

Participants classified as habitual mid-afternoon snackers reported consuming a similar amount of total daily energy compared to participants that were habitual nonsnackers (10184 ± 209 kJ [2434 ± 50 kcal] versus 10389 ± 226 kJ [2483 ± 54 kcal];  *F*(1,191) = 0.61; *P* = 0.44). No significant interactions were observed between habitual snacking status and peanut eating occasion (*P* = 0.64) nor treatment load (*P* = 0.64; data not shown). 

**Figure 2:**
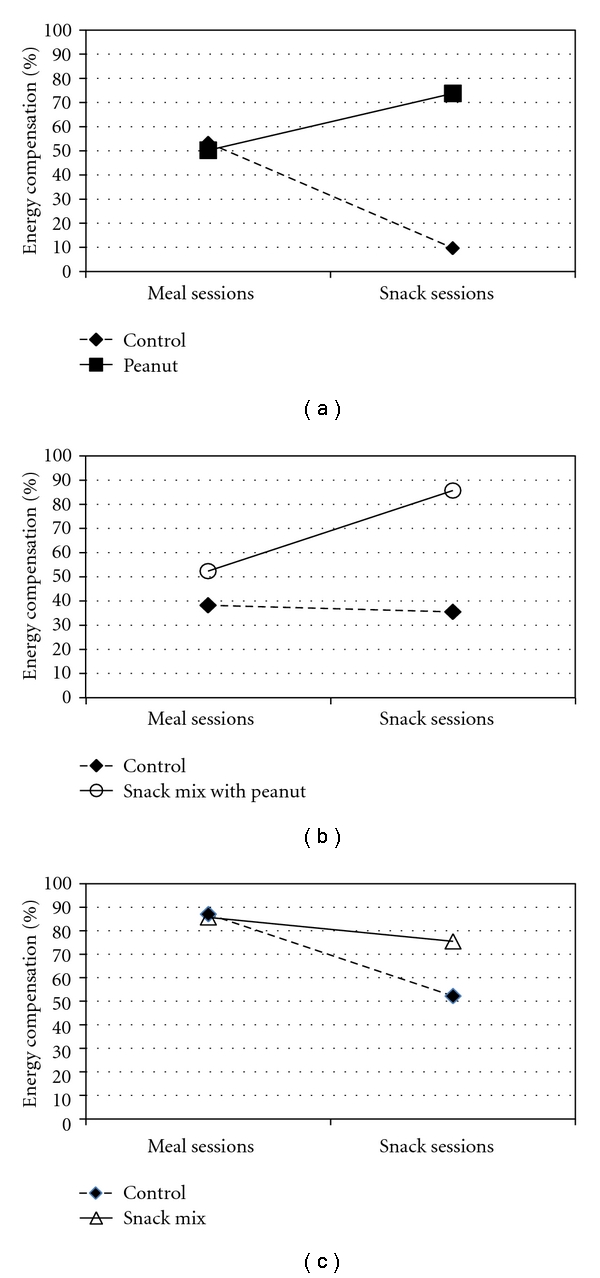
Mean energy compensation observed after ingestion of the test loads. Data are mean values of energy compensation due to the ingestion of the loads.

Energy compensation scores were not significantly different among the three treatments. However, consumption of peanuts, snack mix, and snack mix with peanuts resulted in stronger energy compensation when ingested as a mid-afternoon snack compared to the control snack (Figure 2). The increase in energy compensation was most marked for the peanut load which approached significance *P* = 0.06 and the snack mix with peanut load (*P* = 0.09). Compensation scores were comparable for the experimental loads and the control load when they were ingested with the lunch meal.

### 3.3. Appetitive Ratings

**Figure 3:**
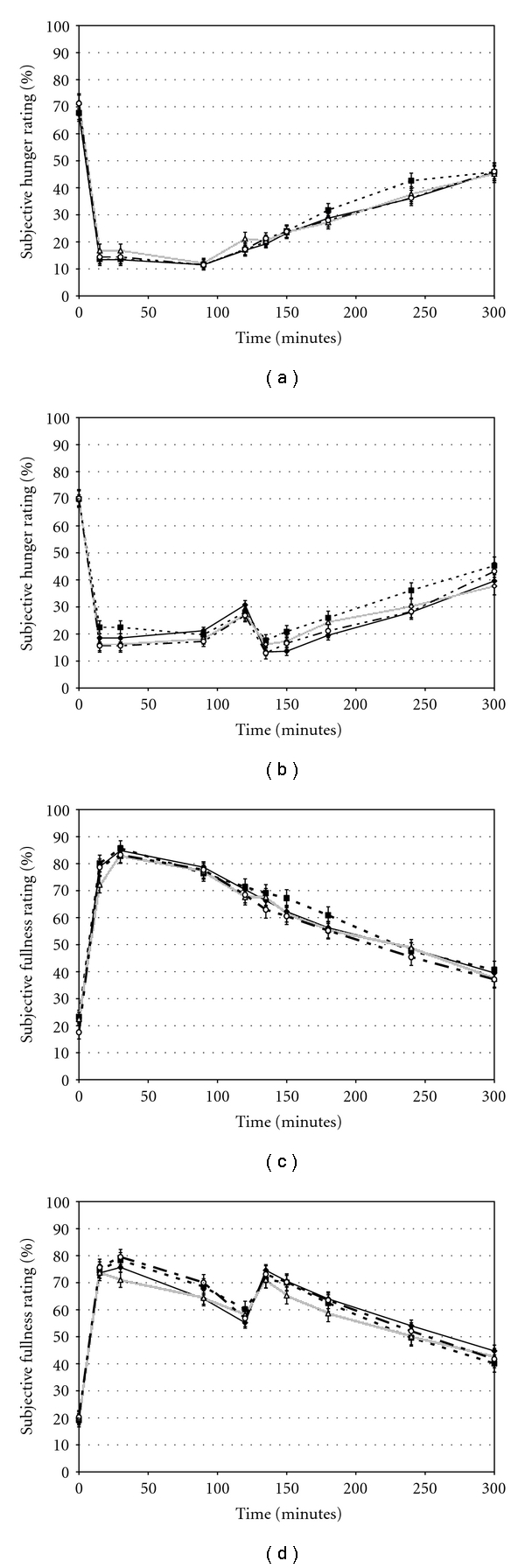
Appetitive ratings for each treatment load. Data are mean (SD) ratings from participants during each session control (triangle), peanut (square), snack mix (diamond), and snack mix with peanuts (circle). Panels (a) and (b) depict hunger ratings from meal and snack sessions, respectively, and fullness ratings are shown in panel (c) for meal sess ions and panel (d) for snack sessions. Peanut *n* = 66; Snack Mix *n* = 68; Snack Mix with Peanut *n* = 67; Control *n* = 201.

Baseline hunger and fullness ratings were not different between treatments for the meal and snack sessions (*F*(3, 596) = 0.40; *P* = 0.75). Average hunger and fullness ratings were compared between peanut, snack mix, snack mix with peanuts, and control at each time point, and no significant trends were observed over time for meal sessions or snack sessions (Figure 3). Hunger and fullness ratings for habitual snackers were not significantly different from those reported by habitual nonsnackers (data not shown).

## 4. Discussion

Recommendations to increase nut consumption due to the potential health benefits they offer must be weighed against their potential to contribute to positive energy balance and exacerbation of overweight/obesity. Evidence that the conditions under which foods are consumed (e.g., state of satiation, time of day) may alter the response they elicit [44, 45, 58, 59] necessitates a consideration of such influences when evaluating the health implications of nut consumption. The present study examined the effects of peanut consumption with a meal or as an afternoon snack (i.e., between 2:00 and 4:00 PM and between customary self-described meals) on appetite and food intake. Responses were contrasted with loads matched on energy, volume, and nutrient composition, but varying in form (i.e., peanuts versus a snack mix) and between individuals who were or were not customary snackers.

### 4.1. Energy Intake by Study Treatment and Timing of Intake

No significant differences in total daily energy intake were observed when 1255.2 kJ (300 kcal) loads of peanuts were ingested compared to loads matched on energy, volume, and macronutrient content (snack mix and snack mix with peanuts treatments) or matched only on energy (control treatment). This finding suggests energy content is an important (but not the sole) determinant of compensation, as the exclusion of other food characteristics retained in the comparative loads, such as masticatory demand [60] was not controlled for in this work. The present study found energy compensation scores for peanut ingestion to range from 50% when peanuts were ingested with a lunch meal to 74% when ingested as a mid-afternoon snack. This is consistent with other published findings that document compensation scores ranging from 55% to 100% for various ground and tree nuts [52, 61–68]. However, the current study suggests that timing of nut ingestion may influence the magnitude of the energy compensation response they elicit. It is notable that only peanut intake resulted in larger energy compensation when ingested as a snack as compared to with a meal. This suggests that the salient attributes of peanuts eliciting energy compensation may be partially masked when peanuts are consumed as a part of a mixed meal. It is possible that the wide range of compensation scores noted in the literature reflect differences in the pattern of nut use as this had not been controlled. 

Although energy compensation was strong, the current experimental design may have attenuated the magnitude of the response by several mechanisms. Participants were required to ingest a standard lunch that provided 30% of their predicted energy needs plus an additional 1255.2 kJ (300 kcal) either with or 120 minutes after the lunch meal (i.e., as a snack). Thus, the lunch was of substantial volume, weight and energy content (2201–4222 kJ [526–1009 kcal]). While it was comparable to reported free-living lunch energy intakes (e.g., 3121–4820 kJ [746–1152 kcal]; [45]), several participants spontaneously commented on the large size of the lunch meal. This was corroborated by a prolonged, over 5 hours, reduction of reported hunger. Many may not have chosen to eat again, but did so 120 minutes after the lunch because of the study design, thus overriding what may have been a compensatory reduction in eating frequency or the energy content of the subsequent eating occasion. Consumption of energy in a “nonhungry” state may reduce the strength or nature of regulatory signals [44, 45]. Additionally, energy consumption sufficiently far ahead of the next eating occasion may also reduce compensation as the satiety value of the load may dissipate before the next spontaneous eating occasion [45]. It is noteworthy, that despite these hurdles, the snack provisions that included *peanuts*  elicited the strongest energy compensation. 

### 4.2. Energy Intake by Habitual Snacking Status

In the US, five commonly self-reported meal patterns have been identified: (1) B, L, D and ≥2 S (2) B, L, D and 1 S (3) B, D and ≥ 2 S (4) B, L, D (5) L, D and ≥ 2 S (where B = breakfast, L = lunch, D = Dinner and S = Snack) [59]. In Brazil the B, L, D and B, L, D, S patterns are most prevalent for adults and adolescents, respectively [69]. To date, no literature has described common meal patterns in Ghana. The current study design assessed two of these patterns as meal (B, L, D) and snack (B, L, D and 1 S) sessions and found similar energy intakes between the habitual snackers and habitual nonsnackers. This finding, while aligned with previous reports [58, 59], is in contrast to the proposed mechanism of snacking leading to increased energy intake and subsequent elevation in BMI. The present findings may reflect the strong satiety property of peanuts [67] and food items possessing similar, yet to be defined, key properties. However, as baseline BMI's did not differ in the discussed published studies it is suggested that “snacker” designation may hold little predictive power for energy balance as compared to the key properties of the snack food item itself. 

### 4.3. Appetite and Study Treatment

Previous work indicated that consumption of food loads matched to peanuts on weight or volume, but not energy, had lesser effects on hunger, as did a semisolid, energy-matched, form of peanut (peanut butter) compared to whole nuts [67]. Foods matched on energy, but varying in nutrient characteristics led to similar effects on hunger. This suggested that energy and rheological properties were important determinants of appetitive responses to food ingestion. The present findings are in agreement with these earlier observations as no differences in appetitive responses were reported following consumption of the peanuts and loads matched on energy content (i.e., the snack mix and snack mix with peanut loads). The comparable response to the energy-matched, but rheologically different control food load, suggests energy is the primary determinant. However, masticatory effort remains a potentially important contributor [60] since the control food still required more oro-mechanical processing than the previously tested peanut butter. 

In summary, this study failed to note acute differential appetitive or compensatory dietary responses from customary mid-afternoon snackers and nonsnackers to food challenges with a mid-day meal or as a mid-afternoon snack. No significant differences in daily energy intake were documented across treatments, but only one postintervention meal was free to vary. This likely limited the detection of treatment effects. Indeed, the percent energy compensation was markedly higher when ingesting a mid-afternoon snack of either peanuts or a snack mix with peanuts relative to the control provision. This is consistent with reports of strong satiety properties for nuts [52, 61–68] and suggestive of an effect of pattern of use. Given some evidence that snacking may disproportionately contribute to positive energy balance [2, 70], further study of the properties of peanuts (and possibly tree nuts) responsible for the stronger compensatory dietary response they elicit when consumed as a snack is warranted as it may provide more general insights related to snack choices in weight management regimens.

## Figures and Tables

**Table 1:** Study treatment session presented to participants.

Treatment	Timing of ingestion	
	Meals	Snacks
Control*	Control provision with lunch meal	Control provisions as an afternoon snack
Experimental: Peanut	Peanut provision with lunch meal	Peanut provision as an afternoon snack
Experimental: Snack mix	Snack mix provision with lunch meal	Snack mix provision as an afternoon snack
Experimental: Snack mix with peanuts	Snack mix with peanuts provision with lunch meal	Snack mix with peanuts provision as an afternoon snack

*All participants completed both meal and snack sessions for the control provision in addition to their randomly assigned experimental meal and snack session for a total of 4 study sessions.

**Table 2:** Composition of the standard study lunch.

Food item	Weight (g)	Total kcal	Fat kcal	Fat (g)	Protein kcal	Protein (g)	CHO kcal	CHO (g)	Fiber (g)
Water	400	0	0	0	0	0	0	0	0
Wheat bread	56	143	21	2	22	5	100	26	4
Cheese^a^	19	65	41	5	16	4	8	2	0
Cooked ham	56	62	18	2	37	9	7	2	0
Mayo^b^	10	71	71	8	0	0	0	0	0
Yellow mustard	5	4	1	0	1	0	2	0	0
Catsup	5	5	0	0	0	0	5	1	0
Carrots	30	12	1	0	1	0	10	2	0
Banana	118	119	5	1	5	1	109	27	4
Chocolate chips^c^	8	43	21	2	2	1	21	5	0

Totals	707	524	179	20	84	21	261	66	8
% Energy			34		16		50		

^a^Kraft American Singles, Northbrook, IL, USA.

^b^Kraft Real Mayonnaise, Northbrook, IL, USA.

^c^Hershey's Milk Chocolate Chips, Hershey, PA, USA.

**Table 3:** Composition of the treatment loads.

Food Item	Water (g)	Fiber^a^ (g)	Cheese^b^ (g)	Potato^c^ (g)	Peanut^d^ (g)	Energy (kJ [kcal])	Fat (%)	Protein (%)	CHO^e^ (%)
Peanut	400	0	0	0	52	1255.2 [300]	70	16	14
Snack mix	400	7	39	15	0	1263.6 [302]	63	19	18
Snack mix w/peanuts	400	4	20	8	26	1330.5 [318]	66	18	16
Control^f^	400	NA	NA	NA	NA	1251.2 [299]	34	16	50

^a^Fiber one cereal, general mills, Minneapolis, MN, USA.

^b^Just the cheese-crunchy baked cheese snack, specialty cheese Co., Lowell, WI, USA.

^c^Potato sticks.

^d^Dry roasted, lightly salted.

^e^Carbohydrate.

^f^The control treatment load was of identical composition to the standard study lunch (Table 2), but adjusted proportionally to provide equal-energy to the other treatment loads.
